# The prevalence of thyroid disorders in COVID-19 patients: a systematic review and meta-analysis

**DOI:** 10.1186/s12902-023-01534-9

**Published:** 2024-01-02

**Authors:** Sadra Ashrafi, Hossein Hatami, Razieh Bidhendi-Yarandi, Mohammad Hossein Panahi

**Affiliations:** 1 Ophthalmic Research Center, Research Institute for Ophthalmology and Vision Science, Shahid Beheshti University of Medical Sciences, Tehran, Iran; 2 Department of Public Health, School of Public Health & Environmental and Occupational Hazards Control Research Center, Shahid Beheshti University of Medical Sciences, Tehran, Iran; 3 Department of Biostatistics and Epidemiology, University of Social Welfare and Rehabilitation Sciences, Tehran, Iran; 4 Safety Promotion and Injury Prevention Research Center, Research Institute for Health Sciences and Environment, Shahid Beheshti University of Medical Sciences, Tehran, Iran; 5 Department of Epidemiology, School of Public Health and Safety, Shahid Beheshti University of Medical Sciences, Tehran, Iran

**Keywords:** COVID-19, Thyroid disorders, Prevalence, Systematic review, Meta-analysis

## Abstract

**Objectives:**

To conduct a systematic review and meta-analysis to evaluate the prevalence of thyroid disorders in COVID-19 patients.

**Data sources:**

Scopus, PubMed, ISI Web of Science, and Google Scholar databases were used in this review. We also consider the results of grey literature.

**Study selections:**

Cohort, cross-sectional, and case-control studies were included.

**Data extraction and synthesis:**

The required data were extracted by the first author of the article and reviewed by the second author. The Pooled prevalence of outcomes of interest was applied using the meta-prop method with a pooled estimate after Freeman-Tukey Double Arcsine Transformation to stabilize the variances.

**Outcomes and measured:**

The different thyroid disorders were the main outcomes of this study. The diseases include non-thyroidal illness syndrome, thyrotoxicosis, hypothyroidism, isolated elevated free T4, and isolated low free T4.

**Results:**

Eight articles were included in our meta-analysis(Total participants: 1654). The pooled prevalence of events hypothyroidism, isolated elevated FT4, isolated low FT4, NTIS, and thyrotoxicosis were estimated (Pooled *P* = 3%, 95% CI:2–5%, I2: 78%), (Pooled *P* = 2%, 95% CI: 0–4%, I2: 66%), (Pooled *P* = 1%, 95% CI: 0–1%, I2: 0%), (Pooled *P* = 26%, 95% CI: 10–42%, I2: 98%), and (Pooled *P* = 10%, 95% CI: 4–16%, I2: 89%), respectively.

**Conclusion:**

Thyroid dysfunction is common in COVID-19 patients, with a high prevalence of non-thyroidal illness syndrome (NTIS) and thyrotoxicosis. Our meta-analysis found a 26% prevalence of NTIS and a 10% prevalence of thyrotoxicosis.

**Systematic review registration:**

PROSPERO CRD42022312601.

**Supplementary Information:**

The online version contains supplementary material available at 10.1186/s12902-023-01534-9.

## Introduction

Since its outbreak in December 2019, COVID-19 has spread widely worldwide and was announced as a pandemic by the WHO in March 2020 [1, 2]. At the end of 2019, the cause of the COVID-19 pandemic was identified to be a new type of coronavirus, known as SARS-COV-2 [3]. According to a previous report from the Chinese Center for Disease Control and Prevention, 14% and 5% of COVID-19 cases were acute and critical, respectively. Also, the death rate of COVID-19 was 2.3% [4].

Caused by SARS-COV-2, COVID-19 has led to a global pandemic. A plethora of COVID-19 survivors experienced a range of symptoms after recovery from the acute form of COVID-19, which is called long COVID, a post-acute sequel of SARS-COV-2, or post-acute COVID-19 syndrome. Long COVID can manifest in the following two forms: (1) symptoms that remain after recovery from the acute phase and (2) new symptoms or syndromes that occur after primary asymptomatic or mild infection. With increasing the population of pandemic survivors, long COVID can result in another pandemic from the existing pandemic. Therefore, it is necessary to identify people who are prone to long COVID [5].

In this context, ACE-2 receptors have been found in various organs, e.g., the cardiovascular, digestive, and endocrine systems, which can cause virus transmission to these organs. In the endocrine system, these receptors are most abundant in the testicles, followed by the thyroid and the hypothalamus. In the thyroid, these receptors make it a suitable target for virus entry [1, 6–8]. Alongside the ACE-2 receptor, the TMPRSS2 receptor also exists on the surface of the thyroid gland, which is a route for the virus entry into the cells [9–11]. The SARS-CoV-2 seems to directly affect the thyroid gland and also the thyroid gland concerning a systemic inflammatory reaction [7, 9]. An autopsy sample taken from the thyroid gland after death showed that COVID-19 directly infected the thyroid gland and caused the dysfunction of these glands [9].

Thyroid disorder as hypothyroidism and NTIS in the acute phase of COVID-19 has been reported in several studies, mainly reporting the improvement of these symptoms during the recovery period of COVID-19. In contrast, a recent article has reported thyroid disorder and autoimmunity during the COVID-19 recovery period [12].

Widespread COVID-19-related thyroid diseases include thyrotoxicosis, hypothyroidism, and non-thyroidal illness syndrome. A change in thyroid function, called non-thyroidal illness, may occur in many acute or chronic clinical conditions. The most common change is a reduction in serum T3 levels, which may be associated with a slight decrease in TSH levels [3, 13]. During acute illness, the T3 hormone level decreases mainly due to decreased deiodinase type 1 enzyme activity, reduced binding of the hormone to thyroid-binding globulin and other binding proteins, and declined TSH levels in acute and long diseases [6]. The total T4 level increases with increasing the severity and duration of NTIS. The intensity of changes in TSH and thyroid hormones is associated with the severity of the underlying NTIS disease. The subsequent changes usually disappear upon eliminating the cause of the disease [3].

Clinical evidence mainly occurs 2–6 weeks after the COVID-19 infection, and patients usually show the prevalent symptoms of thyroiditis, such as pain in the thyroid area. Additionally, drugs such as corticosteroids and heparin used in COVID-19 treatment, interfere with TFT results [6].

SARS-COV-2-related thyroiditis can occur concurrently with COVID-19 or several weeks after recovery. Therefore, it can be inferred that SARS-COV-2 can affect the thyroid either directly (via direct viral effects) or indirectly (through immune system dysregulation). It is noteworthy that some patients experiencing thyroiditis after COVID-19 infection experience a subclinical hypothyroidism phase about 3 months later. Furthermore, Graves’ disease and Hashimoto’s thyroiditis happen several months after subacute thyroiditis, raising the possibility that viral infection may cause autoimmune thyroid disease [14].

The prevalence of thyroid diseases following COVID-19 infection is reported differently in various studies. The prevalence rates of NTIS were reported at 53.7 and 51.7%, respectively, by Dabas et al. and Hashemipoor et al. while this rate is significantly lower (< 10%) in other studies. This difference exists to a lesser extent in the prevalence of other thyroid diseases, such as thyrotoxicosis and hypothyroidism. Moreover, previous systematic reviews have examined thyroid disorders in COVID-19 patients; however, they did not specifically analyze the prevalence of distinct thyroid conditions. Additionally, one such study conducted a systematic review but did not complement it with a meta-analysis to provide a more comprehensive understanding of the data. The knowledge of the most widespread thyroid disease after COVID-19 infection can help the medical staff in early diagnosis and treatment. Therefore, a study with this objective seems to be necessary.

## Methods

### Protocol and registry

This systematic review and meta-analysis utilized the Preferred Reporting Items for Systematic Reviews and Meta-Analyses (PRISMA) guideline as a means of conducting the study (Supplementary file 1) [15]. The present systematic review and meta-analysis was registered in the International Prospective Register of Systematic Reviews (PROSPERO) (CRD42022312601).

### PEO framework

The PEO framework was used to clarify the aim of this research. Accordingly, population (COVID-19 patinets), exposure (COVID-19 pandemic), and outcome (different types of thyroid disorders such as, Hypothyroidism, Isolated elevated FT4, Isolated low FT4, NTIS, Thyrotoxicosis), were included in the systematic review and meta-analysis.

### Search strategy and study selection

In terms of study design, this research includes all cohort, cross-sectional, and case-control studies that investigated the prevalence of at least one thyroid disease in COVID-19 patients. Studies were excluded if they solely reported laboratory data without providing sufficient information to calculate the prevalence of thyroid diseases in the population under study. Additionally, studies focusing on patients with pre-existing thyroid diseases were excluded from the review. There were no restrictions on age, gender, and language in published articles. Scopus, Pubmed, ISI Web of Science, and Google Scholar databases were used in this review. By consulting an expert in this field, the search strategy was designed for Pubmed and used for other databases. The search strategy employed for identifying relevant studies is detailed in the Supplementary file 2.

All articles published from the beginning of the COVID pandemic until December 31, 2022, were included in this review. Furthermore, the references of the obtained articles were reviewed to access more articles. We also consider the results of grey literature.

In the first step, two authors independently reviewed the titles and abstracts of the articles. The full texts of articles that met the inclusion criteria were reviewed by the same two authors. In cases of a discrepancy between two authors in the selection of an article, the final decision was made through a meeting with each other or by reviewing the article by a third author.

### Extraction of data

The required data were extracted by the first author of the article and reviewed by the second author. In case of a mismatch, the third author examined the data and corrected the data. Data were entered into a predesigned electronic checklist by Stata 14.2. The extracted data include:

1. Data on the characteristics of the studies, including the corresponding author's name, the year of publication, the year of study implementation, the publication time, the country of the study implementation, and the number of studied subjects.
2. Data on thyroid disorders, including the type of studied thyroid disorder and the prevalence of each thyroid disorder type (i.e., hypothyroidism, thyrotoxicosis, NTIS, isolated elevated FT4, and isolated low FT4). Also, the AMSTAR 2 checklist was completed to evaluate the study quality (Supplementary file 3) [16].

### Statistical analysis

The statistical analysis was carried out using STATA software (version 14, STATA Inc., College Station, TX, USA). To assess heterogeneity, the Chi-square test and the I-squared index were utilized, with P-values greater than 0.05 and an I-squared value below 50% indicating homogeneity. For detecting publication bias, either Begg’s or Egger’s tests were employed. Meta-prop methodology, enhanced by the Freeman-Tukey Double Arcsine Transformation for variance stabilization, was applied for calculating the combined prevalence of the targeted outcomes. Additionally, forest plots were created to depict each event of interest. Sensitivity analyses were conducted to identify any significantly influential studies. Sensitivity forest graphs were used to display these findings, indicating studies omitted on the left margin and showing the “omitted” meta-analytic summary statistics as a horizontal confidence interval. The overall, or “combined,” results were represented by solid vertical lines. A study’s influence was deemed excessive if its “omitted” analysis point estimate did not fall within the “combined” analysis’s confidence interval. Statistical significance was determined with a threshold of *P*-value less than 0.05.

## Results

### Systematic search results

**Fig. 1:**
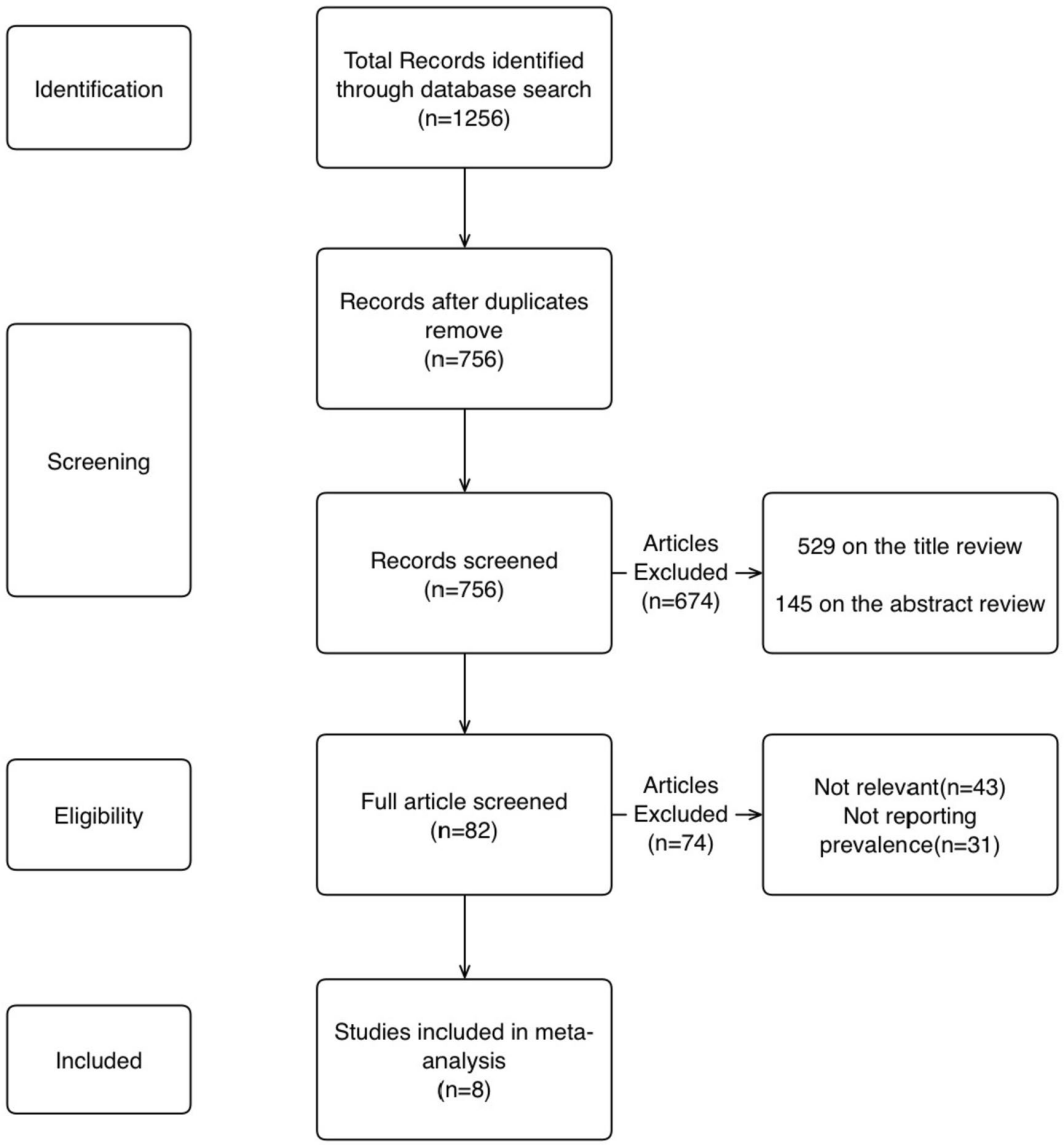
Flow diagram of the study selection process

The initial literature search yielded 1256 studies. The manual search did not add any additional study. In screening for duplication (manually and electronic), 500 were removed. First, the titles were reviewed. by this, 529 studies were excluded. 145 articles were excluded based on the abstract review. After screening, 82 articles were remained. We assessed the full version of these articles. Finally, 8 articles were included in our meta-analysis(Total participants: 1654). A flow diagram of this process is presented in Fig. 1.

The main characteristics of the studies included are summarized in Table 1. Three of these studies were conducted in china, one in Iran, one in Italy, one in Korea an two in India. The COVID-19 patients in these articles were confirmed by reverse transcriptase PCR. The sample size of the included studies was between 100 and 300.

**Table 1:** Summary characteristics of included studies

Author	Country	Published date	Type of study	Number of patients	Female(%)	Median age(years)	NTIS(%)	Thyrotoxicosis(%)	Hypothyroidism(%)	Isolated elevated FT4(%)	Isolated low FT4(%)
Ahhn J, et al.	Korea	2021/3	Cohort	119	47.9	64.3	18.5	14.3	3.4	-	-
Hashemipour S, et al.	Iran	2022/1	Cross-sectional	131	37.9	61	51.7	6.9	6.9	8.1	-
Lania A, et al.	Italy	2020/10	Cohort	287	22.9	66	-	20.2	5.1	-	-
Lui DTW, et al.	China	2021/6	Cohort	122	50.8	58	9.8	4	0.8	1.6	
Lui DTW, et al.	China	2021/9	Cohort	367	53.1	54	7.4	-	-	1.3	0.5
Lui DTW, et al.	China	2021/9	Cohort	204	53.4	55	-	6.3	1.4	0.98	0.98
Dabas, et al.	India	2021/10	Cross-sectional	164	36.3	54	53.7	-	8.53	-	2.1
Chinmay, et al.	India	2021/4	Cohort	251	36.5	-	-	-	1.53	-	-

### Quality assessment

The quality assessment of the included studies is presented in Table 2. In this systematic review, the Joanna Briggs Institute (JBI) Critical Appraisal Checklist was employed as a key tool to assess the quality and validity of the included studies. This comprehensive checklist facilitated a rigorous evaluation of the methodological quality of each study, focusing on aspects such as the suitability of the study design in addressing the research question, potential biases, the reliability of the findings, and the relevance and applicability of the results within the context of our review. On analyzing the 6 cohort studies, the minimum, maximum and median rates of studies classified as “Yes” were 72.7%, 36.3%, 63.6%, respectively. The cohort studies classified as “No” had a maximum report rate of 45.4%, a minimum of 18.1%, and a median of 22.6%. Analysis of the 2 cross-sectional studies showed the report rates classified as “Yes” to be a maximum of 75%, minimum of 62.5%, and median of 68.75%. Those classified as “No” had a report rate of 25%.

**Table 2:** Quality of the studies included in the meta-analysis

Title	1nd Author (y) [ref]	Yes		No		Unclear		Not applicable
		n^a^/N^b^	%	n^a^/N^b^	%	n^a^/N^b^	%	n^c^
Cohort	Ahhn J (2021) [1]	8/11	72.7	1/11	9.1	-	-	2
	Lania A (2020) [17]	7/11	63.6	3/11	27.2	-	-	1
	Lui DTW (2021) [14]	5/11	45.4	5/11	45.4	-	-	1
	Lui DTW (2021) [18]	8/11	72.7	2/11	18.1	-	-	1
	Lui DTW (2021) [12]	7/11	63.6	2/11	18.1	-	-	2
	Chinmay (2021) [19]	4/11	36.3	5/11	45.4	-	-	2
	Max	8	72.7	5	45.4	-	-	-
	Min	4	36.3	2	18.1	-	-	-
	Median	7	63.6	2.5	22.6	-	-	-
Cross-sectional	Hashemipour S (2022) [20]	5/8	62.5	2/8	25	-	-	1
	Dabas (2021) [21]	6/8	75	2/8	25	-	-	-
	Max	6	75	2	25	-	-	-
	Min	5	62.5	2	25	-	-	-
	Median	5.5	68.75	2	25	-	-	-

^a^The number of Joanna Briggs Institute checklist sub-items for cohort, cross-sectional evaluated as “Yes,” “No,” and “Unclear”

^b^Total number of sub-items applicable to the cohort/cross-sectional

^c^Number of sub-items not applicable to the cohort/cross-sectional

### Meta-analyses of the outcomes

**Fig. 2:**
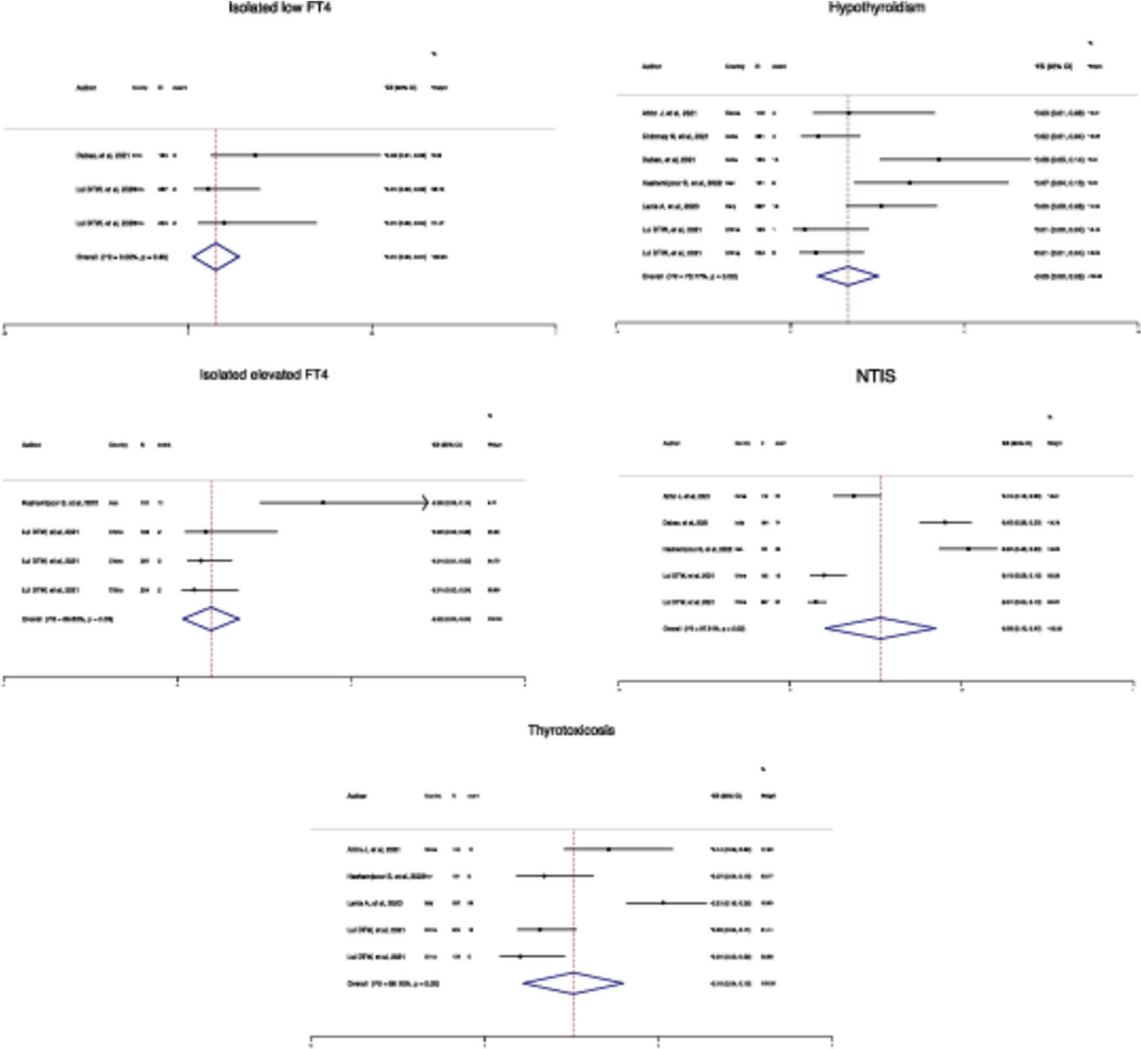
Forest plot of estimated pooled prevalence by various outcomes

The pooled prevalence of events, hypothyroidism, isolated elevated FT4, isolated low FT4, NTIS, and thyrotoxicosis were estimated (Pooled *P* = 3%, 95% CI:2–5%, I2: 78%,), (Pooled *P* = 2%, 95% CI: 0–4%, I2: 66%,), (Pooled *P* = 1%, 95% CI: 0–1%, I2: 0%,), (Pooled *P* = 26%, 95% CI: 10–42%, I2: 98%,), and (Pooled *P* = 10%, 95% CI: 4–16%, I2: 89%,), respectively (Fig. 2).

### Publication bias and sensitivity analysis

**Fig. 3:**
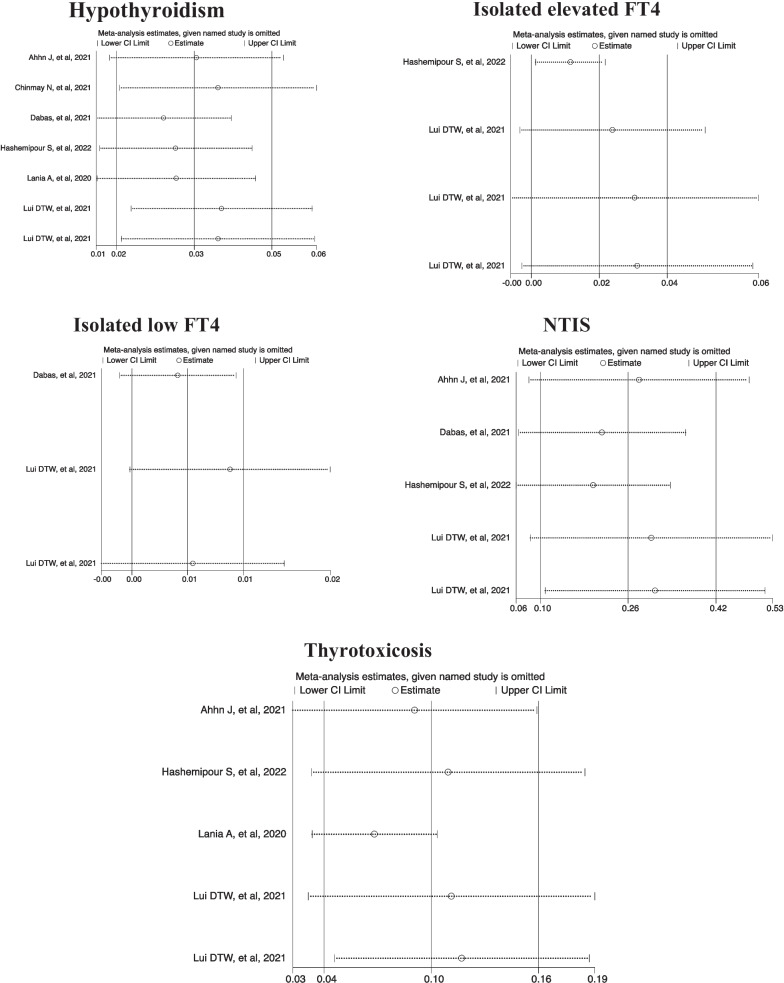
Results of sensitivity analysis

Results of Egger test showed no significant effect of publication bias (*P* value > 0.05) (Table 3). Sensitivity analyses results showed that no single study essentially changed the pooled prevalence of all outcomes (Fig. 3).

**Table 3:** Results of publication bias, heterogeneity and estimated pooled prevalence (95%CI) of the meta-analysis

Outcomes	Publication bias^a^	Heterogeneity (I-squared%)	Pooled prevalence (95%CI)
**Hypothyroidism**	0.167	78%	0.03 (0.02, 0.05)
**Isolated elevated FT4**	0.173	66%	0.02 (0.00, 0.04)
**Isolated low FT4**	0.173	0%	0.01 (0.00, 0.01)
**NTIS**	0.065	98%	0.26 (0.10, 0.42)
**Thyrotoxicosis**	0.143	89%	0.10 (0.04, 0.16)

^a^Egger test

Significant level < 0.05

## Discussion

In this study, the meta-analysis of extracted data represents an estimate of the prevalence of different types of thyroid diseases in COVID-19 patients. Based on the obtained results, the highest and the lowest prevalence rates of thyroid disease belong to NTIS and hypothyroidism, respectively.

The systematic review and meta-analysis conducted by Darvishi et al. have highlighted a significant finding where thyroid disorders were present in 15% of patients with COVID-19, and these disorders were more prevalent in severe cases. Specifically, patients with severe COVID-19 were found to have a 3.77-fold increase in the odds of experiencing thyroid function test (TFT) impairment. Although insightful, the study did not differentiate between the types of thyroid disorders occurring in these patients [22]. In a separate systematic study by Giovanella et al., the majority of COVID-19 patients were euthyroid, with the prevalence of thyroid disorders spanning a wide range from 13 to 64% across various studies. This study, however, did not perform a meta-analysis and did not specify the prevalence rates of different thyroid diseases [23]. These findings indicate a gap in the current understanding and highlight the need for further research into the specific types of thyroid dysfunctions associated with COVID-19.

According to our results, NTIS is most prevalent with a pooled prevalence of 26% among COVID-19 patients. NTIS indicates changes in thyroid hormone levels in severely ill patients in the absence of hypothalamus-pituitary and thyroid disorders. This disease is characterized by decreased levels of T3 and free T4 hormones and an increase in the reverse T3 level. However, the TSH hormone is normal or increases slightly [24].

Dabas et al. reported an NTIS prevalence rate of 53.7% in COVID-19 patients [21]. On the other hand, a prevalence rate of 7.4% was obtained for NTIS by Lui et al. [18]. This difference in the NTIS prevalence rate may result from the severity of COVID-19 in studied subjects. In this regard, Dabas et al. observed the severe form of the disease in 39% of patients [21]. In another study, Lui et al. reported that 75.2 of the subjects suffered from a mild form of the disease, with only 14.4% suffering from a severe form of the disease [18]. The results of this meta-analysis generally indicate a high prevalence of NTIS, despite its different prevalence rates in various studies. Thus, it is necessary to apply some measures for faster diagnosis and control of symptoms.

The second most prevalent thyroid disease in COVID-19 patients is thyrotoxicosis, with a pooled prevalence rate of 10%. The prevalence of thyrotoxicosis was about 20% in a study on COVID-19 patients by Lania et al. They claimed that thyrotoxicosis was more prevalent among COVID-19 patients than among the general population [17]. The prevalence of thyrotoxicosis was between 4 and 15% in other studies [1, 5, 14, 20]. Thyrotoxicosis in COVID-19 patients seems to be a stage of destructive thyroiditis and not a disorder such as Graves’ disease. There are three phases in the course of subacute thyroiditis: (1) thyrotoxicosis during the first few months, (2) hypothyroidism for 3 months, and (3) the euthyroid phase. A reason for this logic is the negative level of antibodies in COVID-19 patients suffering from thyrotoxicosis. However, the negativity of these indicators cannot definitely exclude the incidence of Graves’ disease in these patients [17].

After NTIS and thyrotoxicosis, the third most prevalent thyroid disorder in COVID-19 patients is hypothyroidism, with a pooled prevalence of 3%. Despite a low FT4 in hypothyroidism, the TSH level may be normal. In this condition, the patient will be grouped in the isolated low FT4 category, the prevalence of which is 1% among COVID-19 patients in this study. In COVID-19 patients, hypothyroidism can be central or secondary. The former occurs due to the impaired hypothalamus-pituitary axis, while the latter results from a disruption in the thyroid gland. Hypothyroidism may also occur as a stage of thyroiditis [25]. In a study by Burokovic et al., the number of patients referring to the endocrinology clinic increased significantly in 2022 and 2021 compared to 2019 [26]. In a systematic review, Malik et al. concluded that hypothyroidism was significantly prevalent in COVID-19 patients, who mostly contained low T3 levels along with normal or elevated TSH levels [27].

According to the present results, the pooled prevalence rates are 1% and 2% for isolated low FT4 and isolated elevated FT4, respectively. As shown in different studies, COVID-19 patients may suffer from isolated elevated FT4 in the absence of a specific thyroid disorder, which mainly occurs as a result of NTIS [28]. Hashmipour et al. (2022) documented that approximately 8.1% of COVID-19 patients suffered from isolated elevated FT4 [20]. In three studies conducted by Lui et al., the prevalence of isolated elevated FT4 was between 0.98 and 1.6% [5, 14, 18]. These studies also reported isolated low FT4 in some COVID-19 patients. Although the underlying mechanism of reduced FT4 levels in COVID-19 patients is not fully known, cytokine-dependent inflammations and oxidative stress probably play an important role in suppressing the synthesis and secretion of thyroid hormones. In two studies conducted by Lui et al. [5, 16], the prevalence rates of isolated low FT4 were 0.5 and 0.98% [5, 18]. This value was reported to be 2.1% in the study of Dabas et al. [21].

### Limitations

A major limitation of this review study was the small number of primary articles and, consequently, the size of the primary sample. Fewer studies may exclude the risk of bias or variability in estimates due to limited sample size. Additionally, the limited number of studies may not provide sufficient information to fully explore sources of heterogeneity or to perform subgroup analyses. Accordingly, the validity and generalizability of the results may decrease.

## Conclusion

Thyroid dysfunction is common in COVID-19 patients, with a high prevalence of non-thyroidal illness syndrome (NTIS) and thyrotoxicosis. Our meta-analysis found a 26% prevalence of NTIS and a 10% prevalence of thyrotoxicosis. Clinicians should monitor thyroid function in COVID-19 patients, particularly those with severe illness, and provide appropriate treatment. More research is needed to understand the underlying mechanisms and impact on outcomes.

### Supplementary Information

**Additional file 1.**

**Additional file 2.**

**Additional file 3.**

## Data Availability

The datasets generated and analyzed during the current study are available from the corresponding author on reasonable request.
