# Supplementary material for: The prevalence of thyroid disorders in COVID-19 patients: a systematic review and meta-analysis
Source: BMC Endocr Disord. 2024 Jan 2;24:5. doi: 10.1186/s12902-023-01534-9 (PMC10759498; doi:10.1186/s12902-023-01534-9)
Supplement: Supplementary file 2 — Additional file 2. [file 12902_2023_1534_MOESM2_ESM.docx]

("Hypothyroidism" OR "Thyroid Gland" OR "Thyroid Function Tests" OR "Thyroid Diseases" OR "Hyperthyroidism" OR "Graves Disease" OR "Thyroiditis" OR "Supportive Thyroiditis " OR "Subacute Thyroiditis " OR "Hashimoto Disease" OR "Thyroid Hormones" OR "TSH" OR "T3" OR "T4" OR "De Quervain’s Thyroiditis" OR "Viral Thyroiditis") AND ("Coronavirus Disease 2019 Virus" OR "2019 Novel Coronavirus" OR "2019 Novel Coronaviruses" OR "SARS-CoV-2 Virus" OR "SARS-CoV-2 Virus" OR "SARS-CoV-2 Viruses" OR "2019-nCoV" OR "COVID-19 Virus" OR "COVID 19 Virus" OR "COVID-19 Viruses" OR "SARS Coronavirus 2" OR "Severe Acute Respiratory Syndrome Coronavirus 2" OR "COVID 19" OR "COVID-19 Virus Disease" OR "COVID 19 Virus Disease" OR "COVID-19 Virus Diseases" OR "COVID-19 Virus Infection" OR "COVID 19 Virus Infection" OR "COVID-19 Virus Infections" OR "2019-nCoV Infection" OR "2019 nCoV Infection" OR "2019-nCoV Infections" OR "Coronavirus Disease-19" OR "Coronavirus Disease 19" OR "2019 Novel Coronavirus Disease" OR "2019 Novel Coronavirus Infection" OR "2019-nCoV Disease" OR "2019 nCoV Disease" OR "2019-nCoV Diseases" OR "Coronavirus Disease 2019" OR "SARS Coronavirus 2 Infection" OR "SARS-CoV-2 Infection" OR "SARS-CoV-2 Infection" OR "SARS-CoV-2 Infections")
